# Development of a genome-wide marker design workflow for onions and its application in target amplicon sequencing-based genotyping

**DOI:** 10.1093/dnares/dsac020

**Published:** 2022-08-26

**Authors:** Daisuke Sekine, Satoshi Oku, Tsukasa Nunome, Hideki Hirakawa, Mai Tsujimura, Toru Terachi, Atsushi Toyoda, Masayoshi Shigyo, Shusei Sato, Hikaru Tsukazaki

**Affiliations:** Institute of Vegetable and Floriculture Science, National Agriculture and Food Research Organization (NARO), Tsu, Mie 514-2392, Japan; Tohoku Agricultural Research Center, NARO, Morioka, Iwate 020-0198, Japan; Institute of Vegetable and Floriculture Science, National Agriculture and Food Research Organization (NARO), Tsu, Mie 514-2392, Japan; Kazusa DNA Research Institute, Kisarazu, Chiba 292-0818, Japan; Department of Plant Life Science, Faculty of Agriculture, Ryukoku University, Otsu, Shiga 520-2194, Japan; Plant Organelle Genome Research Center, Kyoto Sangyo University, Kyoto 603-8555, Japan; Plant Organelle Genome Research Center, Kyoto Sangyo University, Kyoto 603-8555, Japan; Comparative Genomics Laboratory, National Institute of Genetics, Mishima, Shizuoka 411-8540, Japan; Laboratory of Vegetable Crop Science, College of Agriculture, Graduate School of Sciences and Technology for Innovation, Yamaguchi University, Yamaguchi City, Yamaguchi 753-8515, Japan; Department of Environmental Life Sciences, Graduate School of Life Sciences, Tohoku University, Sendai, Miyagi 980-8577, Japan; Tohoku Agricultural Research Center, NARO, Morioka, Iwate 020-0198, Japan

**Keywords:** *Allium cepa*, marker design, next-generation sequencing-based genotyping platform, target amplicon sequence, genetic linkage map

## Abstract

Onions are one of the most widely cultivated vegetables worldwide; however, the development and utilization of molecular markers have been limited because of the large genome of this plant. We present a genome-wide marker design workflow for onions and its application in a high-throughput genotyping method based on target amplicon sequencing. The efficiency of the method was evaluated by genotyping of F_2_ populations. In the marker design workflow, unigene and genomic sequence data sets were constructed, and polymorphisms between parental lines were detected through transcriptome sequence analysis. The positions of polymorphisms detected in the unigenes were mapped onto the genome sequence, and primer sets were designed. In total, 480 markers covering the whole genome were selected. By genotyping an F_2_ population, 329 polymorphic sites were obtained from the estimated positions or the flanking sequences. However, missing or sparse marker regions were observed in the resulting genetic linkage map. We modified the markers to cover these regions by genotyping the other F_2_ populations. The grouping and order of markers on the linkages were similar across the genetic maps. Our marker design workflow and target amplicon sequencing are useful for genome-wide genotyping of onions owing to their reliability, cost effectiveness, and flexibility.

## 1. Introduction

Onions (*Allium cepa* L.) are one of the most widely cultivated vegetable crops in tropical, temperate, and boreal regions worldwide. The economic value of onions is derived from their culinary applications, nutritional benefits, and health-promoting properties, such as the presence of flavonoid compounds.^1^ To date, various conventional breeding approaches, including mass selection, inbred line selection, and F_1_ hybrids, have been used to improve yield, quality, and resistance against biotic stresses in each region.^2^ However, to meet the demands of the growing world population in future, it is necessary to enhance breeding efficiency by using advanced breeding technologies.^3^ The utility of molecular markers has been proven by the efficient improvement of target traits through marker-assisted selection (MAS) and genomic selection (GS) in various crops.^4–6^ By contrast, the development and utilization of molecular markers in the breeding of onions have been limited because of their large genome (16 Gb), cross-pollination nature, and high inbreeding depression.^7–10^

Molecular markers, such as random amplified polymorphic DNA, restriction fragment length polymorphism, and simple sequence repeat markers, were initially developed for onion breeding.^11^ These types of markers have been used for genetic diversity estimation, cultivar identification, genetic map construction, and the tagging of chromosome regions affecting traits, such as disease resistance and male sterility.^11^ However, these markers have disadvantages, including a low throughput, low rate of polymorphism among relatives, and considerable time and labour requirements. Recently, single nucleotide polymorphism (SNP) markers have attracted increasing interest because SNPs are abundant, widely distributed throughout the genome, and suitable for high-throughput genotyping.^12^

SNP genotyping platforms are classified as next-generation sequencing (NGS) and array-based technologies. For onions, no SNP bead arrays or chips have been released to date; however, Kompetitive allele-specific polymerase chain reaction (PCR; KASP) and high-resolution melting (HRM) assays have been used for SNP genotyping.^13–15^ KASP assays are simple and yield genotypes with high accuracy and a low missing ratio. In previous studies in onions, KASP-SNP marker sets have been developed using transcriptome data of parental lines and used for the genotyping of a segregating population derived from a cross between the parental lines.^13^^,^^14^ However, the polymorphism rate considerably decreased when the marker sets were used for the genotyping of segregating populations derived from different cross combinations.^13^^,^^14^^,^^16^ Although an optimal marker set should be designed for each plant material, this is costly because KASP primer sets are relatively expensive to design and purchase. HMR assays are also simple, and primer sets for HMR assays are less expensive than those for KASP assays. However, multiplex markers cannot be used in either of these assays. As more markers are used, the assays become more labour- and time-consuming. Thus, flexibility and throughput are potential drawbacks of KASP and HRM assays.

NGS-based genotyping platforms are generally based on genotyping-by-sequencing (GBS), including whole-genome re-sequencing and restriction site-associated DNA sequencing (RAD-seq). These technologies allow simultaneous marker discovery and genotyping at a low genotyping cost. In RAD-seq, regions adjacent to restriction enzyme cut sites across the genome are sequenced and genotyped without the need for *a priori* genomic information. Jo et al.^17^ applied RAD-seq to the genotyping of an onion F_2_ population. Although a highly polymorphic site was detected between the parental lines of the F_2_ population, only ∼2% of the sites remained for genetic map construction after the elimination of unfitting expected segregation and high missing ratio sites. Moreover, because of the low read depth, the possibility of genotyping errors was increased, particularly at heterozygous sites where a high read depth was required for correct genotype calling. Thus, RAD-seq has potential drawbacks, such as genotyping errors, missing data, and the under-calling of heterozygous sites.^18^

Multiplex PCR-based amplicon sequencing methods, including genotyping-in-thousands by sequencing,^19^ multiplex PCR targeted amplicon sequencing,^20^ and highly multiplexed amplicon sequencing,^21^ are other GBS technologies that amplify only the flanking sequences of target SNPs using target-specific primers and incorporate barcode sequence tags into each amplicon. These tags allow the pooling of amplicons derived from each individual into a single sequencing library and classifying individual data after a sequencing run. The advantageous features of amplicon sequencing include a low missing ratio, robustness, low cost per sample, and its applicability to large-genome species, such as common wheat (17 Gb).^22^ Thus, we reasoned that multiplex PCR-based amplicon sequencing may enable high-throughput and cost-effective genotyping in onion breeding.

In previous onion studies, target-specific primer sets, such as KASP and HRM primer sets, were designed based on transcriptome sequences.^13^^,^^15^ Among the designed primer sets, considerable primer sets could not be amplified well when genomic DNA was used as the template. These unamplified primer sets could be designed across splice sites or could bind to multiple non-targeted genomic regions. To design successfully amplified markers, a genomic sequence data is useful. Although onions have a huge genome size, it is possible to obtain draft genome sequences for marker design due to decreasing the NGS sequencing cost. In addition, KASP and HRM markers have been designed without considering genetic or physical map positions. As a result of genetic mapping using these marker genotypes, high-density and sparse marker regions have been detected.^13–15^ Recently, Fujito et al.^23^ reported RNA sequencing (RNA-seq)-based F_2_ population genotyping in onions, allowing the anchoring of more than 4,000 transcriptome sequences (unigenes) on the genetic map. This information is useful for designing markers distributed throughout the genome and allows for cost-effective and informative genotyping.

In the current study, a marker design workflow for onions was developed and applied to a target amplicon sequencing platform. The efficiency of amplicon sequencing was evaluated by genotyping F_2_ populations.

## 2. Materials and methods

### 2.1. Plant materials and DNA and RNA extraction

Two doubled haploid lines (DHA, DHC) derived from a shallot (*A. cepa* L. cv. Chiang Mai) plant and an onion (*A. cepa* L. cv. Sapporoki) plant^24^ were used to construct reference sequence datasets. To generate onion F_2_ segregating populations, the inbred line ‘OPP-6’ (OP6, developed by NARO) and F_1_ hybrid ‘Momiji-3’ (M3, purchased from Shippo Seed Co., Ltd., Kagawa, Japan) were used as parental lines (OP6 and M3 were used as the male and female parent, respectively). The resulting F_1_ plants were grown and self-pollinated to produce F_2_ populations. Three F_2_ populations (F_2__A, F_2__B, F_2__C) derived from different F_1_ plants were used for genotyping. The F_2__A population was grown in 2018, whereas the F_2__B and F_2__C populations were grown in 2019 in the field. Six individuals of OP6 and M3 not used for F_2_ population development were grown in a greenhouse for the detection of polymorphisms between the parental lines. Nuclear DNA was extracted from leaves of DHA by the method reported Lutz et al.^25^ using a DNeasy Plant Mini Kit (Qiagen, Valencia, CA, USA). Total genomic DNA was extracted from leaves of OP6, M3, F_1_, and F_2_ individuals using a DNeasy 96 Plant Kit (Qiagen). Total RNA was extracted from leaves of DHC, OP6, and M3 individuals and the parental F_1_ plants of the F_2_ populations using an RNeasy Plant Mini Kit (Qiagen) or TRIzol reagent (Invitrogen, Carlsbad, CA, USA).

### 2.2. Marker design workflow

**Figure 1:**
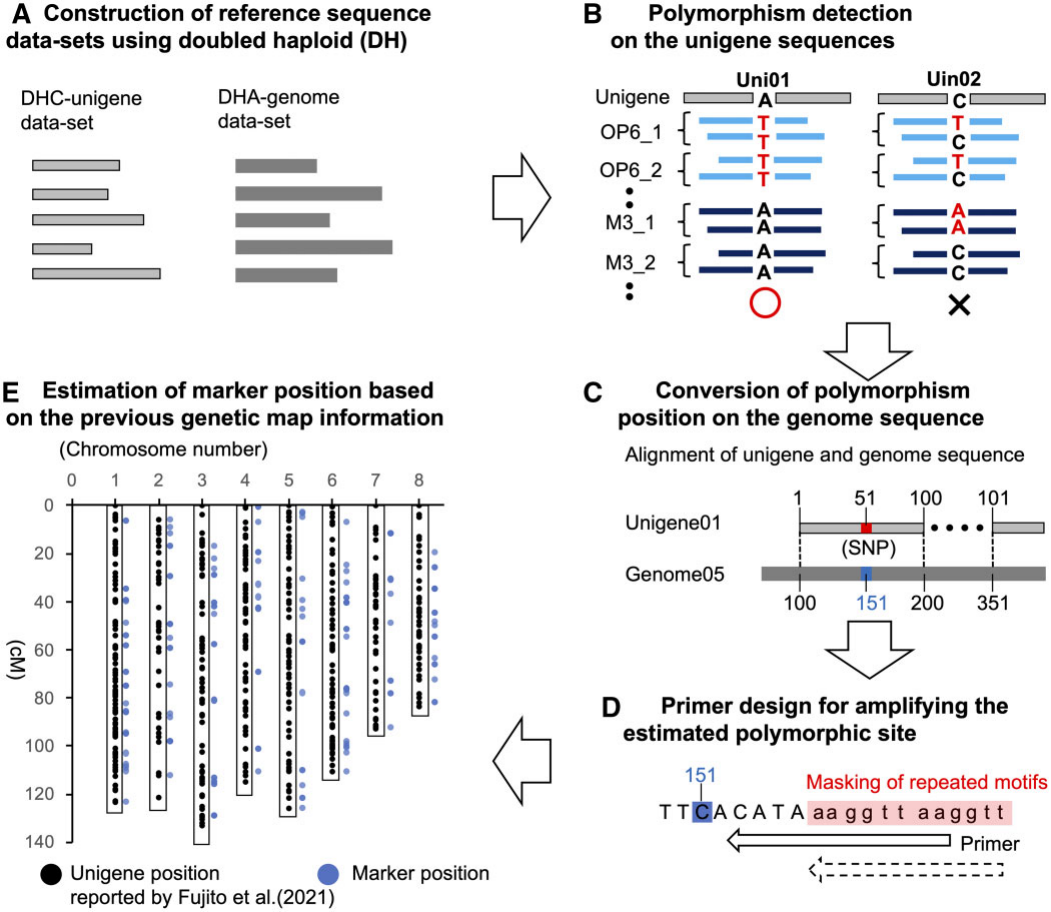
Outline of the workflow used for polymorphism detection among parental lines, primer design, and marker selection.

The marker design workflow was as follows: (i) a reference transcriptome sequence (DHC unigene) dataset and genome sequence (DHA genome) dataset were constructed for polymorphism detection and target-specific marker design; (ii) RNA-seq-based genotyping was performed for individuals of each parental line of the F_2_ populations, and polymorphic sites between the parental lines were detected onto the DHC unigene sequence; (iii) the positions of the polymorphic sites were mapped onto the DHA genome sequence; (iv) target-specific primer sets were designed based on the DHA genome sequence to amplify the estimated polymorphic sites; and (v) the positions of the markers on the genetic map or linkage groups (LGs; chromosome number) were estimated based on correspondence with the sequences reported by Fujito et al.,^23^ and markers covering the whole genome were selected (Fig. 1).

### 2.3. Reference unigene and genome sequence construction

The cDNA library was generated using a SureSelect Strand-specific RNA Library Prep System (Agilent Technologies, Senta Clara, CA, USA) according to the manufacturer’s instructions and was sequenced using the Illumina NextSeq 500 platform, generating 75-base single-end reads. Reads including adapter sequences, more than 5% unknown nucleotides, and more than 20% low-quality nucleotides (QV ≤ 10) were excluded using fastx_clipper in FASTX-toolkit v0.0.14 (http://hannonlab.cshl.edu/fastx_toolkit/). The remaining reads were assembled into contigs using Trinity v2.5.1 (https://github.com/trinityrnaseq/trinityrnaseq/releases). In total, 55,025 contigs were obtained with an average length of 1.04 kb and N50 length of 1,639 bp. The average length of the constructed unigene set (the DHC unigene set) was approximately twice that of the DHA unigene set reported by Fujito et al.^23^ (Supplementary Table S1).

Information about the LG (chromosome number) or genetic map position was assigned to each DHC unigene based on information reported for the corresponding DHA unigene by Fujito et al.^23^ Using the DHA and DHC unigene sequences as ‘query’ and ‘subject’, respectively, BLAST searches were performed using blastn in NCBI’s BLAST+ (version 2.6.0), and best-matching DHA unigene information was assigned to each DHC unigene (Supplementary Table S2).

To generate a draft genome sequence, a whole-genome sequencing library was prepared using a TruSeq DNA PCR-Free Library Prep Kit (Illumina, San Diego, CA, USA) and sequenced on an Illumina HiSeq 2500 platform, generating 250-bp paired-end reads (insert size of 600 bp). The adaptors were trimmed off using fastx_clipper in FASTX-toolkit v0.0.14. Nucleotides with a QV <10 at the 3′ terminus were trimmed off using PRINSEQ v0.20.4.^26^ Reads including Ns were excluded. After quality trimming, reads of 250 bp were selected and classified into paired and single reads. The paired reads were assembled using SOAPdenovo2 rev240, with a kmer size of 127.^27^ The scaffolds were linked with the paired reads using GapCloser v1.10 (https://sourceforge.net/projects/soapdenovo2/files/GapCloser/) with default parameters.

The genome size was estimated to be 16,595,919,574 bp using Jellyfish, with a kmer size of 17.^28^ The total and N50 lengths of the contigs were 16,137,143,537 and 3,470 bp, respectively, whereas the total and N50 lengths of the scaffolds were 15,601,554,442 and 15,475 bp, respectively. The completeness of the genome assembly was assessed by Benchmarking Universal Single-copy Orthologs (BUSCO)^29^ v3.0.2; database version embryophyta odb10. The BUSCO scores were as follows: single copy (68.6%), duplicated (2.8%), fragmented (13.8%), and missing (14.8%).

### 2.4. Transcriptome sequence analysis

RNA-seq of the individuals of each parental line was performed on a HiSeq Transcriptome platform at BGI. The adaptors and barcodes were trimmed off, and low-quality reads (< Q20) were eliminated from the raw data at BGI. Further analysis was performed using CLC Genomics Workbench v11.0 (Qiagen). Clean data for each individual sample were mapped to the DHC unigene set, and local re-alignment and duplicate mapped read removal were conducted with default parameters. Variants were called using fixed ploidy variant detection setting. The minimum coverage, minimum count, and minimum frequency were set at 5%, 2%, and 20%, respectively. Variant sites with an allele frequency of >80% or <80% were redefined as ‘homozygous variants’ and ‘heterozygous variants’, respectively.

### 2.5. Design of target-specific markers

Polymorphic sites between parental lines at which a homozygous variant was fixed in one parental line, but not detected in the other parental line, were extracted from the variant calling data (Fig. 1B). To avoid primer set design across splice sites (intron–exon junctions), the positions of polymorphic sites on the DHC unigene sequence were mapped onto the DHA genome sequence by blastn, using the DHC unigene and DHA genomic scaffold sequences as ‘query’ and ‘subject’, respectively. The best alignment results were used to estimate the positions of polymorphic sites on the DHA genome sequence (Fig. 1C, Supplementary Table S2). Primer sets to amplify the estimated polymorphic site were designed using the GENOMEMASKER package.^30^ First, to avoid designing primers binding at multiple loci, repeated sequence motifs were searched on the DHA genome sequence and listed in a blacklist file using the glistmaker programme.^30^ Because the programme is not adapted to the multi-FASTA format (i.e. DHA genome scaffolds file), DHA genome scaffolds were randomly divided into 21 groups and connected with 50 ‘N’ insertions between scaffolds within each group (in total, 21 FASTA files were created), and these files were used to prepare the blacklist file. Second, for use as a reference for marker design, the upper and lower flanking 150-bp sequences of the estimated polymorphic sites were extracted and listed in a file (a total of 300 bp was listed per estimated polymorphic site). Using the blacklist and the sequence file, repeated sequence motifs on sequences were masked, and forward and reverse primer sets were designed to produce 120–160-bp amplicons including the estimated polymorphic sites using the gmasker and gm_primer3 programmes^30^ (Fig. 1D). The numbers of primer binding sites and possible PCR products were checked using the gtester programme.^30^ Based on this information and reported genetic map information,^23^ primer sets were designed to amplify specific loci covering the whole genome (480 primer sets in total). The forward and reverse primers were tailed with the common sequence tags, CS1 (5′-ACACTGACGACATGGTTCTACA-3′) and CS2 (5′-TACGGTAGCAGAGACTTGGTCT-3′), respectively, to allow for the addition of adaptor and barcode sequences during a second round of PCR according to the protocol reported by Ishikawa et al.^22^

### 2.6. Library preparation

For library preparation, we used the protocol reported by Ishikawa et al.,^22^ with some modifications. The 480 primer sets for genotyping of the F_2__A population were divided into 10 primer mixes using the MultiPLX programme.^31^ Thus, each primer mix comprised 48 forward and reverse primers. The reaction mix for the first PCR had a total volume of 10 μl and comprised 1× Multiplex PCR Master mix (Qiagen), a primer mix, and 10–60 ng template DNA. The first-PCR thermal cycling programme included an initial denaturation step at 95°C for 15 min followed by 32 cycles of 30 s at 94°C, 90 s at 60°C, and 1 min at 72°C, and a final extension at 72°C for 10 min. One microliter of the first-PCR products derived from the same template DNA was collected and mixed (10 μl in total), and the pool of first-PCR products was purified using Agencourt AMpure XP Reagent beads (Beckman Coulter) according to the manufacturer’s protocol. The purified PCR products were suspended in 100 μl of 0.1× TE buffer and diluted 100 times with distilled water, and 1 μl of each dilution was used as a template for the second PCR. As described by Ishikawa et al.,^22^ bidirectional amplicon tagging was performed in the second PCR. The 10-μl second-PCR mix contained 1× Multiplex PCR Master mix (Qiagen), 400 nM forward and reverse fusion primers, and template. The second-PCR thermal cycling programme was as follows: 95°C for 15 min followed by 10 cycles of 30 s at 94°C, 90 s at 62°C, and 30 s at 72°C. Five microliters of the second-PCR products derived from the same template was pooled (10 μl per sample), and size selection was conducted using Agencourt AMpure XP Reagent beads as follows. In a first step, large DNA fragments were removed. Ten microliters of the pooled second-PCR products, 10 μl of low TE buffer, and 14 μl of AMpure XP Regent were mixed well. After a 5-min incubation at room temperature, the samples were placed onto a magnetic separator for 5 min, and the supernatants were collected and transferred to new tubes. In a second step, small DNA fragments were removed. Five microliters of AMpure XP Reagent was added to the supernatants and mixed well. After a 5-min incubation at room temperature, the samples were placed onto a magnetic separator for 2 min, and the supernatants were discarded. The beads with the PCR products attached were washed twice with 200 μl of 70% EtOH. Finally, the purified PCR products were suspended in 40 μl of low TE buffer. The concentrations of the purified second-PCR products were measured using a Wallac 1420 Arvo Mx Multilabel Counter (PerkinElmer) and Quant-iT PicoGreen dsDNA Assay Kit (Invitrogen). The volume of each sample was adjusted to 2 ng of DNA (amplicon), and the samples were then pooled into a single library. The quality of the library was assessed using an Agilent 2100 Bioanalyzer and High-Sensitivity DNA kit (Agilent Technologies). The region covering all PCR library peaks was measured and used to calculate the DNA concentration of the library, which was then adjusted to 75 pM by dilution. Sequencing was performed using the Ion Torrent Proton system and an Ion PI Chip (Thermo Fisher Scientific). For genotyping of the F_2__B and F_2__C populations, 345 primer sets selected from the 480 primer sets and an additional 96 primer sets were randomly divided into eight and two primer mixes, respectively (in total, 10 primer mixes were used for library preparation). To reduce non-uniform marker amplification, the above protocols were modified as follows: the number of cycles was reduced from 32 to 25 in the first PCR, the purified first-PCR product was not diluted, and 1 μl of purified product was directly used as the template for the second PCR.

In total, four libraries were prepared and sequenced. The first library contained 63 F_2__A, three OP6 and M3, one F_1　_(F_1__A), and the other individuals (these were not related to the materials mentioned above and not used for genotyping analysis in this study); in total, 96 samples were pooled in a single library. The second and third libraries included 192 samples, and the fourth library included 96 samples. Two-hundred and forty-five F_2__B individuals were assigned to the second and fourth libraries, whereas 204 F_2__C individuals were divided over the third and fourth libraries. Three OP6 and M3 individuals and two F_1_ plants (F_1__B, F_1__C) were duplicated and included in the third and fourth libraries.

### 2.7. Data processing and genotype calling

Removal of the Ion Torrent sequencing adaptor sites and common sequence tags (CS1, CS2) and demultiplexing of the barcodes to separate the different samples were performed using Torrent Suite v5 (Thermo Fisher Scientific). The trimmed sequence data were mapped to the DHA genome sequence using Torrent Suite v5. Mapping results derived from the same barcode were merged using SAMtools.^32^ Valliant calling was performed using Torrent Variant Caller with default germline low-stringency parameter settings (Thermo Fisher Scientific). Base positions expected to correspond to polymorphisms were designated as ‘hotspots’, and aligned reads in the hotspots were counted.

### 2.8. Genetic linkage map construction

To generate a genetic map of the F_2_ population, first, the consensus genotypes of the parental lines were manually determined for each polymorphic site using three parental plants, because some polymorphic sites were not fixed in the parental lines. Using the consensus genotypes, the genotype of each F_2_ was converted from reference/alternative code (1, 0, –1) to parental code (A, H, B). Genotypes with >10% missing data or segregation distortion (*P *<* *0.001) were removed.

Linkage maps of each F_2_ population were constructed using JoinMap v4.0.^33^ The logarithm of odds thresholds used for the grouping of DNA markers was >4.0 (F_2__A population) or >10.0 (F_2__B and F_2__C populations). The marker order was determined using the maximum likelihood mapping algorithm. The recombination frequency was converted into genetic distance (cM) using the Haldane mapping function. JoinMap v4.0 was used to reveal synteny between genetic maps. LGs were assigned to chromosomes according to a previous report.^23^

## 3. Results

### 3.1. Detection of polymorphisms between the parental lines

To detect polymorphisms between the parental lines, RNA-seq-based genotyping was performed using several individuals of each line. The mapping ratio of each sample was ∼80%, except for OP6_1 (47%; Supplementary Table S3). The OP6_1 sample was removed because of a low mapping ratio, and data for the other samples were used for variant detection. Among the total variant sites, homozygous variant sites accounted for 42–47% and 34–36% in OPP6 and M3 individuals, respectively (Supplementary Table S4). Because the genotypes of individuals of a same line were not fixed in onions, the homozygous variant sites shared among the individuals of same parental line were counted (Supplementary Table S5). In total, 43,528 (25%) homozygous variant sites were observed in at least four out of five OP6 individuals, whereas 38,851 (27%) sites were shared by at least five out of six M3 individuals (Supplementary Tables S5 and S6). These variant sites were defined as fixed homozygous variant sites of each parental line, and the polymorphic sites in which the variants were not detected in the individuals of the opposite parental line were extracted from these fixed homozygous variant sites. Thus, 4,642 and 3,509 sites were identified as polymorphic sites in OP6 and M3, respectively (8,151 sites in total, Supplementary Table S6). These polymorphic sites were classified into three categories based on information reported by Fujito et al.^23^ (Table 1). Polymorphic sites for which positions on the genetic map could be estimated were denoted as ‘anchored’, sites for which only the chromosome number was known were denoted as ‘chromosome-only’, and all others were denoted as ‘no information’.

**Table 1:** Classification of polymorphic sites and marker selection

	Anchored on the genetic map			Identified only in the chromosome			No information	
Chromosome	No. of polymorphisms	No. of unigenes	No. of markers	No. of polymorphisms	No. of unigenes	No. of markers	No. of polymorphisms	No. of unigenes
1	162	35	31	205	66	20	–	–
2	285	50	40	395	91	30	–	–
3	138	30	25	222	48	37	–	–
4	99	19	19	392	66	42	–	–
5	169	22	20	393	68	42	–	–
6	156	32	31	156	38	29	–	–
7	148	25	21	259	59	41	–	–
8	154	24	22	355	61	30	–	–
Total	1,311	237	209	2,377	497	271	4,463	1,004

Polymorphic sites were classified into three categories based on information reported by Fujito et al.^23^

### 3.2. Target-specific primer design

Based on the polymorphic site information, markers for genotyping of the F_2_ segregation population were designed. To avoid primer set design across splice sites (intron–exon junctions), the positions of polymorphic sites on the DHC unigene sequence were mapped onto the DHA genome sequence using blastn. Each primer set was designed to amplify an estimated polymorphic site, and 480 markers were selected from the anchored and chromosome-only markers to cover the whole genome (Table 1, Supplementary Fig. S1).

### 3.3. Genotyping by multiplex PCR-based amplicon sequencing

**Figure 2:**
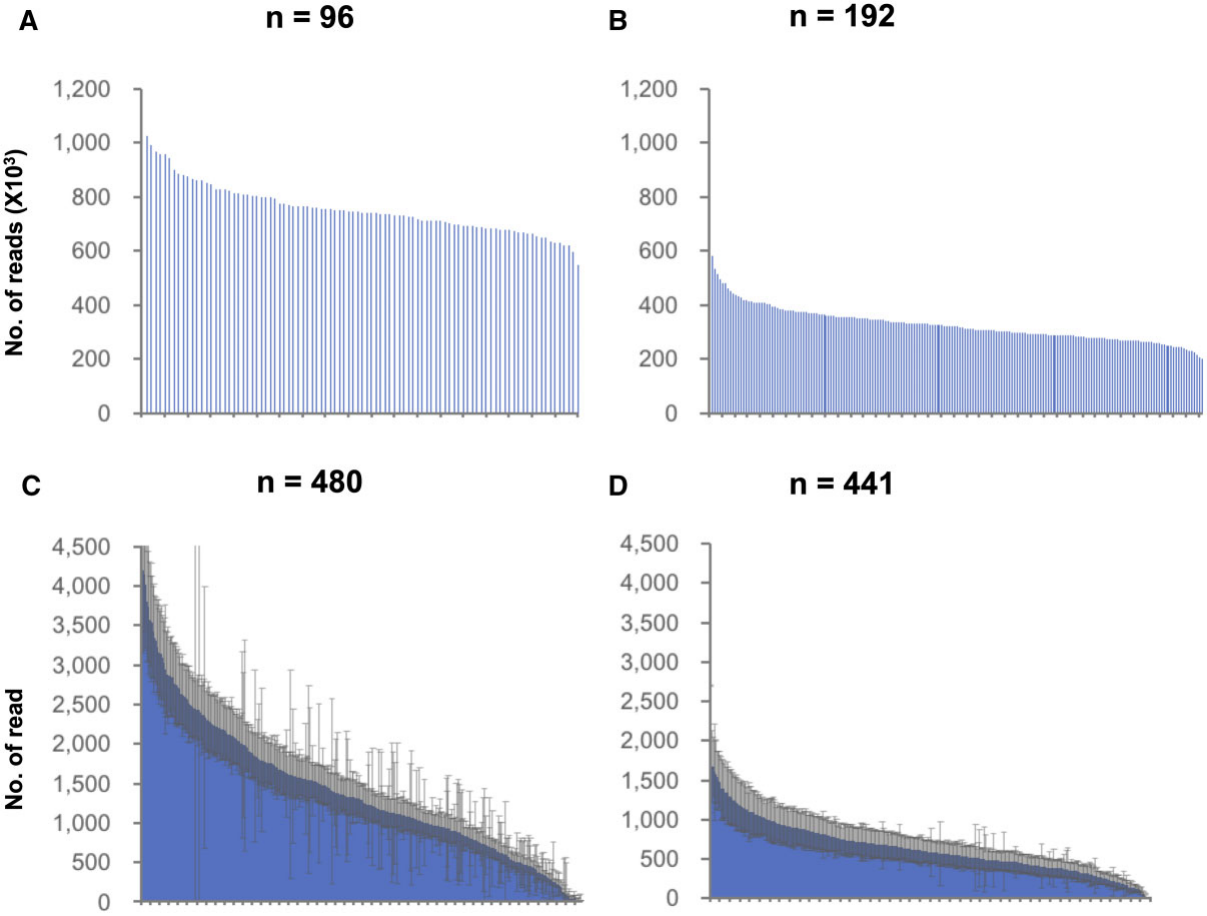
Read number variation among samples and hotspot sites detected by amplicon sequencing. (A and C) Amplicon sequencing results for the first library. (B and D) Amplicon sequencing results for the second library. (A and B) Total read number per sample, (C and D) average read number per marker (hotspot). Error bars in (C) and (D) indicate standard deviations for 96 and 192 samples, respectively.

The numbers of reads per sample and per marker for the first library are shown in Fig. 2A and C. The number of reads per sample was relatively stable, and the average number of total reads was 709,404. However, the number of reads per marker was highly variable. In addition, some markers showed an extremely large standard deviation (Fig. 2C). To validate the genotyping efficiency, first, the genotypes of ‘hotspots’ in each marker were compared between the parental lines. The markers were classified into five groups as presented in Table 2. Two-hundred and fifty-four markers (52.9% of the total) showed polymorphisms between the parental lines. There were 91 markers with no variants (19.0%) and 93 unfixed markers (19.4%). Next, markers denoted as ‘heterozygous’ at the hotspots were counted in F_1__A (Table 2). In total, 283 (59.0%) heterozygous hotspots were found in F_1__A.

**Table 2:** Classification of markers based on the mapping results for each hotspot site

		Validation using parental lines		No. of hotspots designated as ‘heterozygous’ in F_1__A plant	
Class	Description	No. of hotspots	% in total	No. of hotspots	% in total
1	Polymorphism detection^a^	254	52.9	230	47.9
2	No variant^b^	91	19.0	–	–
3	No call^c^	3	0.6	–	–
4	Genotyping only one parent^d^	39	8.1	–	–
5	Unfixed^e^	93	19.4	53	11.0
	Total	480	100.0	283	59.0

a Genotypes were fixed among individuals of each parental line, and polymorphisms were detected between the parental lines.

b There was no variant.

c Sufficient coverage for genotyping was not obtained.

d Genotypes of individuals of one parental line are indicated as ‘no call’ owing to insufficient coverage of amplicons.

e Genotypes were not fixed among individuals of each parental line.

### 3.4. Marker selection for genetic linkage map construction

As a result of mapping and genotype calling, new polymorphic sites that differed from the hotspot sites were detected among Class 2 markers (no variant in hotspots, Table 2). Based on this information, polymorphic sites for genetic map construction were selected according to the following criteria: (i) sites designated as ‘heterozygous’ in F_1__A were extracted from all polymorphic sites, (ii) when more than two polymorphic sites were detected in one amplicon sequence, the most representative one (e.g. hotspot) was selected. In total, 329 polymorphic sites (markers) including SNPs, multiple nucleotide polymorphisms (MNPs), and insertions/deletions (Indels) were selected (69% of 480 markers, Supplementary Table S7).

**Figure 3:**
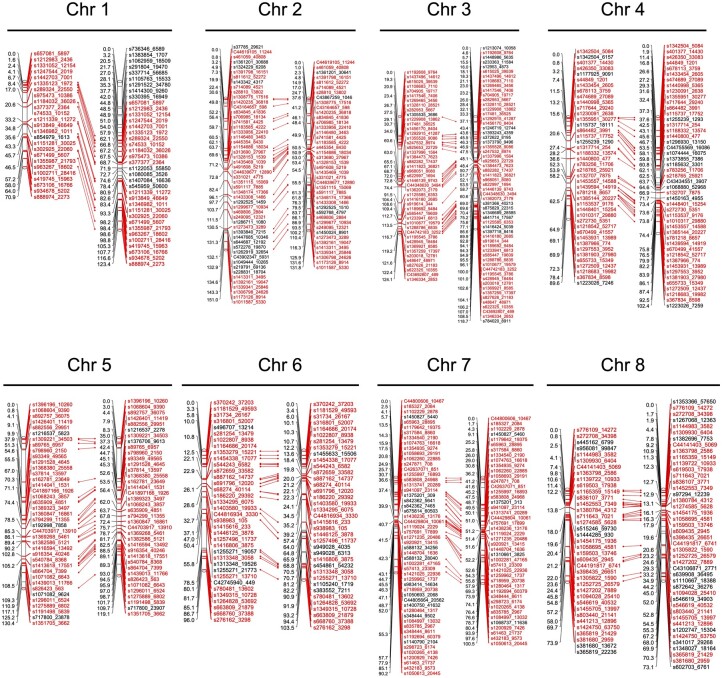
Comparison of the genetic linkage maps of the F_2__A and F_2__B populations. Linkage groups on the left and right in each chromosome were derived from the F_2__A and F_2__B population, respectively. Common polymorphic sites (markers) segregating in both populations are in red font, and red lines indicate the correspondence of a relative marker position between two linkage groups.

A genetic linkage map was constructed for 63 F_2__A using the 329 markers (left LGs in Fig. 3, Supplementary Table S8). The resulting linkage map comprised eight LGs and covered a total of 826 cM (Supplementary Table S9). The length of LG1 corresponding to Chr1 was 71 cM (Fig. 3, Supplementary Table S9), which was ∼50 cM less than that reported previously^23^ because of the lack of markers located in the upper part of Chr1.

### 3.5. Marker set development

A marker set was developed to improve the genotyping efficiency and cover the missing genome regions, including the upper part of Chr1. First, several markers that could not amplify the target sequence and capture the polymorphic sites between the parental lines were eliminated from the 480 markers, and the remaining markers were arranged according to the following criteria: (i) when more than two markers were located on the same DHA genome scaffolds and all of them were used for F_2__A genetic linkage map construction, a representative one was selected and the others were removed; (ii) markers that captured the polymorphism between the parental lines, but did not segregate in the F_2__A population were excluded, because these markers may segregate in an F_2_ population derived from different F_1_ plants. As a result, 318 and 37 markers were selected from the former and latter criteria, respectively. Thus, in total, 345 markers were selected from the 480 markers. Second, 96 markers were added to cover the sparse and missing genetic regions. Using RNA-seq data obtained from F_1__B and F_1__C plants, sites designated as ‘heterozygous’ were identified, and markers were selected to cover the sparse and missing regions (Supplementary Fig. S1). In total, 441 markers were designed and used for genotyping of the F_2__B and F_1__C populations (Supplementary Table S7).

### 3.6. Validation of genotyping using the developed markers by amplicon sequencing

The numbers of reads per sample and per marker for the second library are shown in Fig. 2B and D. The variation in the number of reads per sample was similar to that in the first library, whereas the variation in the number of reads per marker was lower. To validate the efficiency of the developed markers, the number of hotspot genotypes designated as ‘heterozygous’ was counted in F_1_ plants. Among the 345 markers selected from the original 480-marker set, 289 and 294 markers showed heterozygous genotypes in F_1__B and F_1__C. Among the additional 96 markers, 42 markers showed heterozygous genotypes in both F_1__B and F_1__C. In total, 331 (75%) and 336 (76%) markers were designated as ‘heterozygous’ in F_1__B and F_1__C, respectively. Only four markers showed ‘No call’ in both F_1__B and F_1__C. Except for hotspots, polymorphic sites were detected in several markers, some of which showed heterozygous genotypes in F_1_ plants. For genetic map construction, polymorphic sites were selected as described above. In total, 349 and 342 polymorphic sites (markers), including SNPs, MNPs, and Indels, were selected in the F_2__B and F_2__C populations (79% and 78% of the 441-marker set), respectively (Supplementary Table S7). Three-hundred and seventeen polymorphic sites were common in both populations.

Genetic linkage maps were constructed using the F_2__B and F_2__C population genotypes (Fig. 3, Supplementary Fig. S2 and Table S8). The resulting linkage map comprised eight LGs (eight chromosomes) and covered 871 and 859 cM in the F_2__B and F_2__C populations, respectively (Supplementary Table S9). In both maps, the length of Chr1 was more than 50 cM longer than that in the map of F_2__A because of the additional markers located in the upper part of Chr1 (Fig. 3, Supplementary Table S9). The grouping and order of markers on the linkages were quite similar between the F_2__A and F_2__B populations (Fig. 3), which was confirmed upon comparison of the F_2__B and F_2__C populations (Supplementary Fig. S2).

## 4. Discussion

In the current study, we developed a marker-design workflow for onions. The characteristics and usefulness of this workflow are as follows. First, transcriptome-based genotyping was used to search the polymorphisms between parental lines. Transcriptome-based genotyping is a cost-effective approach for rough identification of polymorphisms among whole-genome regions, particularly in organisms with a large genome size.^15^^,^^34^^,^^35^ Second, a unigene dataset was used as a reference for transcriptome-based mapping and genotyping. Although a DHA genome dataset was also constructed in this study, this dataset was not suitable as a reference sequence for transcriptome-based mapping and genotyping owing to its huge sequence size. If the genomic sequence dataset was used as a reference, considerable computational resources and calculation times would be required. Tanaka et al.^35^ compared the efficiency of RNA-seq-based mapping and genotyping in barley when the whole genome (4.8 G) or transcribed region (0.59 G) was used as a reference sequence. Most samples had a better mapping ratio for transcribed regions than the whole genome, and the procedure using transcribed regions rather than the whole genome reduced the calculation time by two thirds. As also described by Tanaka et al.,^35^ unigene datasets may be useful for efficient transcriptome-based mapping and genotyping in onions. Third, the positions of polymorphic sites in unigenes were mapped to the genomic sequence, and target-specific primers were designed based on the genomic sequence. Almost all primer sets were amplified well and could be used for genotyping. Only three primer sets could not be sufficiently amplified among 480 primer sets designed in this study (‘No call’ in Table 2). In a previous study, 326 primer sets were not amplified well among 1,256 KASP-SNPs primer sets designated based on the transcriptome sequence.^13^ These unamplified primer sets would be designed across splice site or bind multiple non-targeted genomic regions. To increase the number of successfully amplified primer sets, the procedure for primer design was based on the genomic sequence. Fourth, the positions of the markers on the genetic map were estimated based on previous high-density linkage map information, and markers covering the whole genome were selected. Our selected markers were distributed throughout the whole genomic region with low redundancy and sparse region coverage (Fig. 2). Accordingly, the genetic maps in the current study were better than those of previous studies in which higher numbers of markers were used.^13^^,^^15^ Thus, this marker selection procedure is useful for reducing the number of markers, enabling cost-effective genotyping.

We applied our marker-design workflows to a target amplicon sequencing platform; however, the workflow may be useful for other genotyping platforms, such as KASP and HRM marker-design. Additionally, it is possible to change the reference sequence in our marker design workflow. A recent study released the sophisticated onion genome sequence, which was built using Illumina short-reads, PacBio long-reads, and Dovetail scaffolding.^36^ Thus, using the released genome sequence in our marker-design workflow may increase the number of markers successfully capturing the polymorphisms.

In the current study, multiplex PCR-based amplicon sequencing was applied to the genotyping of onion populations. As only the target genome regions were amplified and sequenced, a relatively high read depth per target site was achieved (Fig. 2). The corresponding ratio of genotypes between the duplicated samples was more than 97% (Supplementary Table S10). Genetic linkage maps of the F_2_ populations could be constructed with a low missing ratio (Supplementary Table S9), and the positions and order of markers (polymorphic sites) were similar among the F_2_ populations (Fig. 3, Supplementary Fig. S2). These results suggested that this genotyping platform had a high robustness. The high read depth makes amplicon sequencing advantageous over other NGS-based genotyping platforms such as RAD-seq owing to the decreased rates of missing ratios and genotyping errors, particularly at heterozygous sites. Moreover, amplicon sequencing has a higher throughput than KASP and HRM assays. Multiple samples and markers can be analysed simultaneously per sequencing run in amplicon sequencing, whereas such multiplexing cannot be performed in either KASP and HRM assays. In this study, 192 samples with 441 amplicons were sequenced and genotyped at the same time. Additionally, amplicon sequencing has high flexibility, i.e. markers can be easily rearranged in an amplicon sequencing platform. Markers that cannot amplify the target locus and capture polymorphisms were removed, and new markers were added to cover the sparse and missing genome regions. These features of amplicon sequencing are beneficial for the genotyping of real-life materials and the implementation of genotype-based selection methods, such as MAS and GS, in onion breeding programmes.

Next, we designed the marker set based on polymorphic site information from the parental lines. First, the sites for which the genotypes were fixed among the lines were detected using transcriptome data of six individuals (Supplementary Table S5), and polymorphic sites between the parental lines were searched by comparing the fixed sites (Supplementary Table S6). However, onion lines are highly heterozygous; thus, the fixation rate within the lines was low (Supplementary Tables S4 and S5), which makes it difficult to design primer sets in some regions (Supplementary Fig. S1). In addition, because the success rate of marker capture at the expected polymorphic sites was rather low, sparse and missing genome regions were found in the genetic linkage map (left in Fig. 3). The transcriptome data of the parental F_1_ plant were very useful to design new markers covering such regions (Supplementary Fig. S1). Although the parents of the F_1_ plant were not able to be genotyped in this study, transcriptome data from the parents would have been useful to design primer sets to capture polymorphisms in the progeny population given the high heterozygosity of onion lines.

We used the high-density genetic map information reported by Fujito et al.^23^ for marker selection (Fig. 1E, Table 1). The lengths of genetic distances on each chromosome in the current study were equivalent to those on the high-density genetic map reported by Fujito et al.^23^ (Supplementary Tables S8 and S9), suggesting that this information may be useful for designing marker sets covering the whole genome without redundancy, thereby contributing to cost-effective genotyping. The marker set covering the whole genome may also be useful for capturing quantitative trait loci and estimating the genetic value of each individual, which contributes to efficient breeding based on MAS and GS. Moreover, several markers selected from the chromosome-only marker class were anchored on the genetic linkage maps in the current study (Supplementary Table S2). Using such marker information, the positions of 751 DHA unigenes belonging to the chromosome-only class reported in Fujito et al.^23^ could be estimated based on the similarity between the DHA unigene, DHC unigene, and DHA genome sequences (Supplementary Table S2). This information will also be beneficial for efficient marker design in the future.

When the first library, including the F_2__A population, was sequenced, the read numbers were relatively uniform among samples, but variable among markers (Fig. 2A and C). By contrast, in the second library, there were fewer amplification differences among markers, although the primer sets and numbers of samples used differed from those in the first library (Fig. 2B and D). Thus, this observation may be explained mainly by the fact that the cycle number was reduced in the first PCR. A uniform amplification of markers contributes to low missing and genotype error rates, particularly at heterozygous sites where at significant read depth is required for correct genotype calling. Thus, an optimized protocol is beneficial for the genotyping of a segregating population and breeding materials. For some markers, the standard deviation was extremely large (Fig. 2C), likely because these markers amplified a sequence from only one parental line (categorized as Class 3 in Table 2). This may be because of polymorphisms present in the 3′ end of a primer binding site, and further optimization of marker design should allow us to avoid this issue.

In the current study, a marker set was developed to capture polymorphisms between particular Japanese onion lines. By comparing the amplicon sequences with the flanking sequences used for KASP-SNP marker design reported by Duangjit et al.,^13^ only 7 of 1,256 flanking sequences showed similarity with the amplicon sequences in a blastn search (Supplementary Table S7). This high number of new polymorphic sites highlights the importance of polymorphism analysis for each material of interest. Onion varieties have been developed to be adapted to the cultivation environment in different regions. For example, long-day, intermediate-day, and short-day onion varieties have been developed and are used in different regions according to their daylength requirement for bulb formation. The germplasm used in breeding programmes differs among cultivation regions and thus, marker sets should be optimized for the genetic resources used in each breeding programme. As custom primer sets are less expensive in design and purchase and more flexible and more reliable in use than primers used in some existing methods, were conclude that amplicon sequencing is suitable for application in practical onion breeding programmes.

## Supplementary data

Supplementary data are available at DNARES online.

## Supplementary Material

dsac020_Supplementary_Data

## Data Availability

Raw data of the DHC RNA-Seq and the DHA genome sequences are available in the DDBJ under accession No. DRA012913 and BQKJ010000001-BQKJ012832894, respectively. Both assembled information has been published in the Allium Transcriptome Database (http://alliumtdb.kazusa.or.jp).

## Accession numbers

DDBJ numbers: DRA012913, BQKJ010000001-BQKJ012832894.
