# Supplementary figures and images for: Development of a genome-wide marker design workflow for onions and its application in target amplicon sequencing-based genotyping
Source: DNA Res. 2022 Aug 26;29(5):dsac020. doi: 10.1093/dnares/dsac020 (PMC9410872; doi:10.1093/dnares/dsac020)

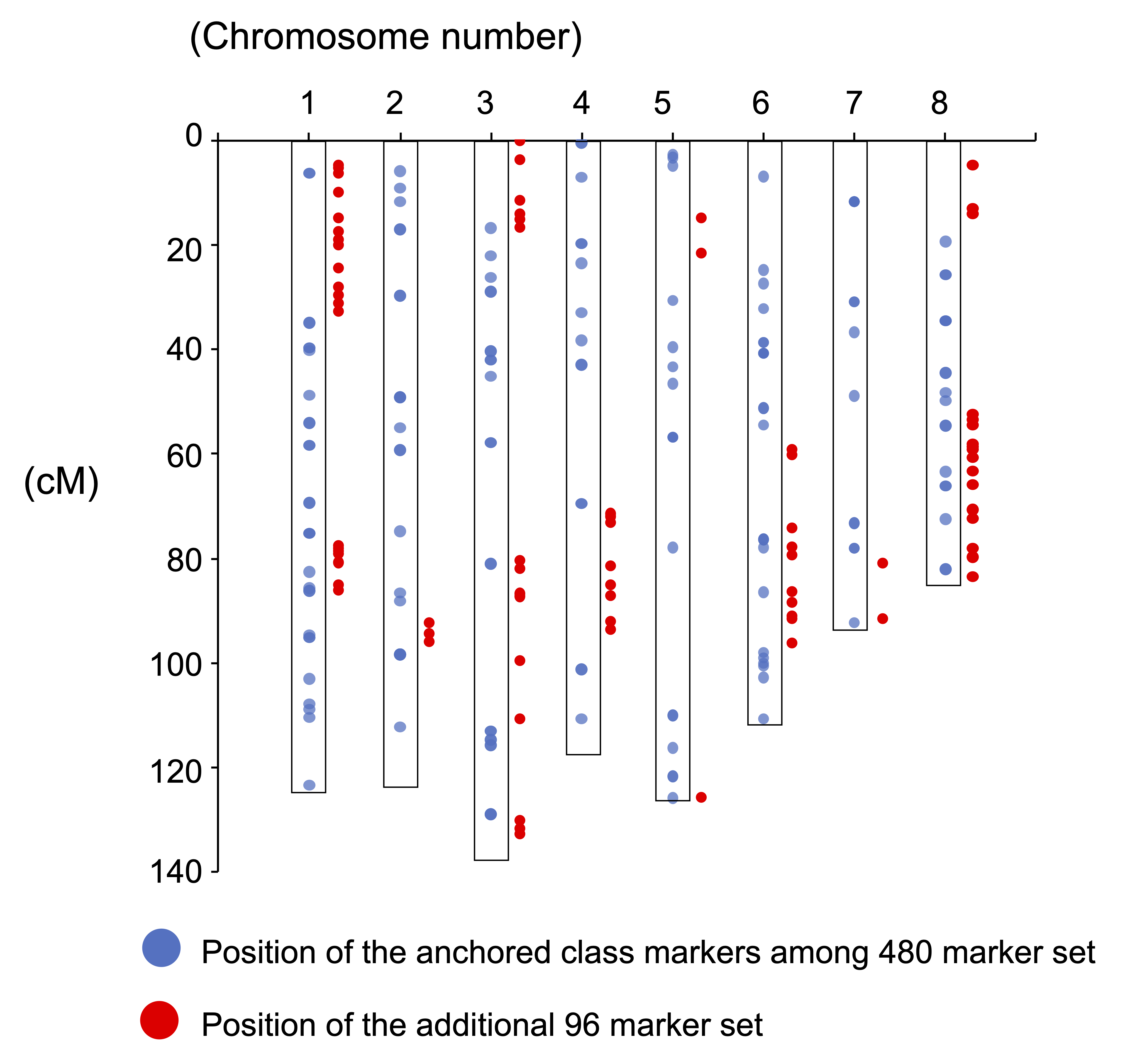

Supplement: dsac020_Supplementary_Data [file dsac020_supplementary_data.zip › Sup_Fig1.tiff]
